# Promoting technology-enabled primary eye care in South-East Asia

**Published:** 2022-03-01

**Authors:** Taraprasad Das, Yuddha Dhoj Sapkota

**Affiliations:** 1 Vice-Chairman: LV Prasad Eye Institute, Hyderabad, India.; 2 Regional Coordinator South-East Asia: International Agency for Prevention of Blindness, Kathmandu, Nepal.

**Technology is a useful aid in reaching low-cost and effective primary eye care to a larger population.**

## Prioritising primary health care

In 2018, Member States of the WHO reaffirmed the criticality of prioritising primary health care to ensure that people receive comprehensive promotive, preventive, curative, rehabilitative, and palliative care as close as feasible to their everyday environments.^1^^,^^2^ They pledged to use, with community participation, methods and technology that are practical, scientifically sound, socially acceptable, easily accessible, and affordable.^1^

## Primary eye care and integrated people-centred eye care

**Figure 1:**
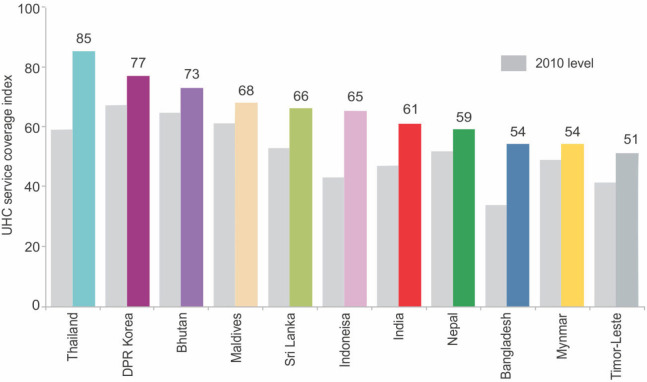
Changes in coverage of essential health services, 2010–2019^3^

The essential health services index for universal health coverage in South-East Asia has increased from an average of 46% in 2010 to an average of 61% in 2019 (Figure 1).^3^ Though essential health service coverage has continued to improve in the region since 2010, projections point to the need for an accelerated rate of improvement across all health components to reach the goal of good health and well-being (Sustainable Development Goal 3) by 2030.^3^ The four essential health service components are (i) reproductive, maternal, neonatal, and child health; (ii) infectious diseases; (iii) non-communicable diseases; and (iv) health service capacity and access.

In this context, primary eye care is a vital component of primary health care and universal health coverage. It includes the promotion of eye health, the prevention and treatment of conditions that may lead to visual loss, and the rehabilitation of those already blind. Primary eye care is the primary health care approach to the prevention of blindness.^4^ In 2019, the WHO released the *World Report on Vision*. One of its important recommendations was ‘integrated people-centred eye care’ (IPCEC). In 2020, the World Health Assembly resolved to urge the Member States ‘to implement integrated people-centred eye care in health systems’; ‘to make eye care an integral part of universal health coverage’; and ‘to promote high-quality implementation and health systems research complementing existing evidence for effective eye care interventions’.^5^ The resolution also urged Member States to focus on effective interventions for two common avoidable and treatable eye disorders—refractive error and cataract.

Eye care is planned for as part of the essential services provided at primary health centres and is best delivered by trained eye health personnel in the primary health care system.^6^ For instance, primary eye care is delivered exclusively through the national primary health care system in Bhutan. In some other countries of South-East Asia, i.e., Bangladesh, India, and Nepal, a hybrid model is prevalent, with the public health care system and non-governmental organisations (NGOs) both providing primary eye care.

Primary eye care centres are equipped to correct refractive errors and identify common eye diseases in all countries, particularly cataract, diabetic retinopathy, glaucoma, and corneal infection, for appropriate referrals. These centres can advise the public on measures to avoid corneal injury and help rehabilitate people with low vision through community-based rehabilitation programmes.^7^

## Status of primary eye care in South-East Asia

Primary eye care can be delivered through a fixed facility or a mobile facility at the community level. Primary care facilities are designed to provide primary eye care only. In many countries in the region, primary health centres lag behind standalone centres in providing primary eye care. Table 1 shows the status of primary eye care in 2019 in South-East Asia (only available data are included). While we do not have data from the entire region, the available data indicate that a good network of facilities exists for primary eye care in Bangladesh, India, and Nepal in the NGO sector.

**Table 1:** Status of primary eye care in South-East Asia, 2019

**Country**	**Government facilities**			**Large NGO facilities**				
	**No. of PHCs** *	**Number of districts in the country**	**No. of patients examined** **	**No. of eye care organisations**	**Vision centres**	**Patients seen at mobile facilities**	**Patients seen at fixed facilities**	**Total number of eye patients examined**
**Bangladesh**	70	64	170,745	10	150	-	1,083,750	1,254,495
**Bhutan**	33	20	763,092	-	-	-	-	763,092
**India**	30,045	718	328,992,750	9	425	2,664,568	1,862,713	4,527,281
**Indonesia**	-	7,024	-	-	-	-	-	-
**Maldives**	-	Atolls 17	-	-	-	-	-	-
**Myanmar**	-	76	-	-	-	-	-	-
**Nepal**	203	77	-	32	170	-	-	1,327,603
**Sri Lanka**	-	25	-	-	-	-	-	-
**Thailand**	9,826	878		-	-	-	-	-
**Timor-Leste**	14	13	18,238	-	-	2,306	-	20,544

* PHCs—primary health centres.

** This is the general pool of patients, including those needing eye care. Specific eye care data are not available.

## New technology in eye care

It is increasingly realised that technology can help in providing primary eye care to a larger population, especially those in remote locations. The use of technology enables the delivery of low-cost and quality eye care at the doorstep in three ways: remote screening and diagnosis, real-time delivery of treatment, and monitoring of continued care.^8^ Technology-enabled portable handheld devices, such as the device for refraction, slit lamp, and fundus camera, can be easily used at a fixed facility (vision centre/primary health centre) or even, in the future, for a home-based eye examination. These devices allow a vision technician to provide comprehensive care through external eye examination (using a slit lamp), prescription of spectacles (to correct refraction errors), performing of a field test (for evaluation of glaucoma) and capturing fundus images (for evaluation of the retina) in diverse settings—primary eye care facility, school, or home.

Two other new technologies are live teleconsultation and artificial intelligence (using machine and deep learning) for diagnosis. Today's smartphone-based technology allows for teleconsulting in both synchronous mode (live video-conferencing) and asynchronous mode (store-and-forward video-conferencing). The technology of artificial intelligence is gaining ground as an aid in medical diagnosis.

Although using technology involves some inherent costs, the cost-benefits are many: among them, optimal utilisation of clinical facilities and resources, better/more efficient patient consultation by care providers, and reduced cost of consultation for patients.^8^ Technology will continue to advance. And we will have to take technology into account for all health-related ‘future planning’ and ‘responsive care’. In this process, especially with healthier and happier patients in mind, it is very important to integrate technology with local culture.^9^
